# Modern physiology vindicates Darwin's dream

**DOI:** 10.1113/EP090133

**Published:** 2022-08-14

**Authors:** Denis Noble

**Affiliations:** ^1^ Department of Physiology Anatomy & Genetics University of Oxford Oxford UK

**Keywords:** Charles Darwin, evolutionary biology, extracellular vesicles, George Romanes, inheritance of acquired characteristics, John Burdon‐Sanderson, pangenesis

## Abstract

**New Findings:**

- **What is the topic of this review?**
  Revisiting the 2013 article ‘Physiology is rocking the foundations of evolutionary biology’.
- **What advances does it highlight?**
  The discovery that the genome is not isolated from the soma and the environment, and that there is no barrier preventing somatic characteristics being transmitted to the germline, means that Darwin's pangenetic ideas become relevant again.

**Abstract:**

Charles Darwin spent the last decade of his life collaborating with physiologists in search of the biological processes of evolution. He viewed physiology as the way forward in answering fundamental questions about inheritance, acquired characteristics, and the mechanisms by which organisms could achieve their ends and survival. He collaborated with 19th century physiologists, notably John Burdon‐Sanderson and George Romanes, in his search for the mechanisms of transgenerational inheritance. The discovery that the genome is not isolated from the soma and the environment, and that there is no barrier preventing somatic characteristics being transmitted to the germline, means that Darwin's pangenetic ideas become relevant again. It is time for 21st century physiology to come to the rescue of evolutionary biology. This article outlines research lines by which this could be achieved.

## 1 HISTORICAL INTRODUCTION: DARWIN'S PHYSIOLOGICAL COLLABORATIONS

This article revisits an earlier article published in *Experimental Physiology* nearly a decade ago (Noble, 2013) entitled ‘Physiology is rocking the foundations of evolutionary biology’. The justification for a revisit is that many new physiological experiments and interpretations of genomic data have appeared. The time is ripe for a reassessment.

I begin with a largely ignored historical fact: Charles Darwin's later ideas on evolution inspired new physiological experimentation on the processes that could be involved. He was also deeply involved in those experiments. In fact, in the last decade (1872–1882) of his life, he collaborated with three physiologists: Michael Foster, John Burdon‐Sanderson and George Romanes. These collaborations initially focused on the physiological processes that could explain some of Darwin's observations on plants at his home, Down House in Kent.

He was intrigued by plants capable of catching insects, such as Venus’ fly‐trap, *Dionaea muscipula*. The leaves develop rows of sensitive hairs which sense when an insect arrives on the plant. What intrigued Darwin was the rapidity with which the convex leaves can snap together while changing shape to become concave, so forming a cavity within which the insect becomes trapped (Hodick & Sievers, 1988). Plants are not generally capable of such rapid movement. The fly‐trap and similar insectivorous plants are unusual in reacting so quickly. He worked therefore with Burdon‐Sanderson to determine whether the rapid trigger might be electrical, just as 19th century physiologists had demonstrated rapid action potentials in nerves and muscles in animals. Burdon‐Sanderson (1873, 1888) and Burdon‐Sanderson and Page (1876) showed that the mechanism does indeed involve an action potential (Williams, 1973, 2002). Modern experiments show that plants do this via calcium channels (Beilby, 1984; Williamson & Ashley, 1982; see Krol et al., 2006 for discussion and further references). (Darwin did not put his name to the 1873 publication, as was his custom generally in such collaborations.)

Burdon‐Sanderson also introduced Darwin to his student at UCL, George Romanes, which led to a collaboration of great importance to evolutionary biology. In *The Origin of Species* (Darwin, 1859) Darwin had already subscribed to the inheritance of acquired characteristics through use and disuse, in addition to the process of natural selection. He refers to such inheritance around 12 times in the book. In his Introduction to the 1964 Harvard reprint of Darwin's book (Mayr, 1964, 1982), Mayr writes:

Curiously few evolutionists have noted that, in addition to natural selection, Darwin admits use and disuse as an important evolutionary mechanism. In this he is perfectly clear. For instance… on page 137 he says that the reduced size of the eyes in moles and other burrowing mammals is ‘probably due to gradual reduction from disuse, but aided perhaps by natural selection’. In the case of cave animals, when speaking of the loss of eyes he says, ‘I attribute their loss wholly to disuse’ (p. 137). On page 455 he begins unequivocally, ‘At whatever period of life disuse or selection reduces an organ…’. The importance he gives to use or disuse is indicated by the frequency with which he invokes this agent of evolution in the Origin. I find references on pages 11, 43, 134, 135, 136, 137, 447, 454, 455, 472, 479, and 480.

Nine years later, in *The Variation of Animals and Plants Under Domestication* (Darwin, 1868), he speculated on the possible mechanisms of pangenesis since he realised that, in organisms with separate specialised germ‐lines, there would need to be communication between the soma and the germ‐line for such pangenetic inheritance to be possible. He treated his theory of pangenesis as a 'beloved child’ (Desmond & Moore, 1991, p 551), so this was no passing fancy. He very much wished it to be true. He postulated the existence of tiny particles, which he called gemmules, which could communicate from the soma to the germ‐line. He wrote:

Physiologists maintain, as we have seen, that each cell, though to a large extent dependent on others, is to a certain extent, independent or autonomous. I go one step further, and assume that each cell casts off a free gemmule, which is capable of reproducing a similar cell. (Darwin, 1868, vol. 2, pp. 377)

He fully acknowledged the speculative nature of his theory:

The existence of free gemmules is a gratuitous assumption, yet it can hardly be seen as very improbable, seeing that cells have the power of multiplication through the self‐division of their contents. (Darwin, 1868, vol. 2, pp. 378)

He therefore imagined his gemmules as rather like spores. As I will show in a later section of this article, Darwin was correct to see cells as ‘casting off a free gemmule’, and we have had to wait for more than a century for the resolution of microscopy of living tissues to become great enough to visualise what I will argue are Darwin's gemmules (see video ‘Rediscovering the real Darwin’: https://www.youtube.com/watch?v=H8jPyHFKU7I).

But, in orthodox 20th century evolutionary biology, Darwin's idea was dismissed outright since, if true, it would break a cardinal, but unproven (see, e.g., Noble, 2016, pp. 126–128), assumption of the Modern Synthesis, that is, the Weismann Barrier, which postulates that the germline is isolated from influences via the organism or its environment. It is important to note that Weismann's idea was first formulated after Darwin's death in 1882 (Weismann, 1892, 1893). Darwin therefore never had an opportunity to respond to Weismann's radical proposal.

Yet, the evidence shows that, had he lived to see it, Darwin would have opposed Weismann, since Darwin treated pangenesis as his ‘beloved child’, in the sense that he put a lot of effort into trying to prove it. This evidence is clear in his sustained collaboration with George Romanes. Their strategy was to perform experiments in which the tissues of different plant species were grafted together to see whether they could communicate their presumed gemmules, and so their characteristics, to each other, conceivably even fusing to form new species. Had they succeeded, they would have discovered a mechanism by which hybridisation could lead to a form of symbiogenesis.

Romanes became Darwin's staunch defender against Weismann. When Darwin passed away, Romanes persisted with the experiments, and eventually published an article in the *Zoological Journal of the Linnean Society* in which he proposed a theory of physiological selection in addition to natural selection (Romanes, 1886). Romanes also became the Secretary of the Linnean Society. But his theory of physiological selection remained just that, an interesting and potentially ground‐breaking theory, but largely without the experimental evidence that he and Darwin had tried hard to find. The problem was that the methods of microscopy of the 19th century did not have the resolution required to visualise what might have existed as the postulated gemmules.

Romanes died in 1894, at the early age of 46. Had he lived just another few years he would have witnessed the rediscovery of Mendel's work on genetics and could have planned pangenesis experiments much more likely to succeed. He might even have predated Waddington (1942, 1959) in his fruit fly experiments showing the inheritance of an acquired characteristic. As it was, Darwin's dream that his young colleague might vindicate his pet theory died with Romanes. It would take more than a century before that dream could be fully resurrected. Unfortunately, Weismann and his imagined Barrier, not Romanes's and Darwin's also‐imagined gemmules, became the basis on which the 20th century Modern Synthesis was developed. Romanes's and Darwin's ‘beloved child’ was still‐born.

## 2 DARWIN AND THE FOUNDATION OF THE PHYSIOLOGICAL SOCIETY IN 1876

**FIGURE 1:**
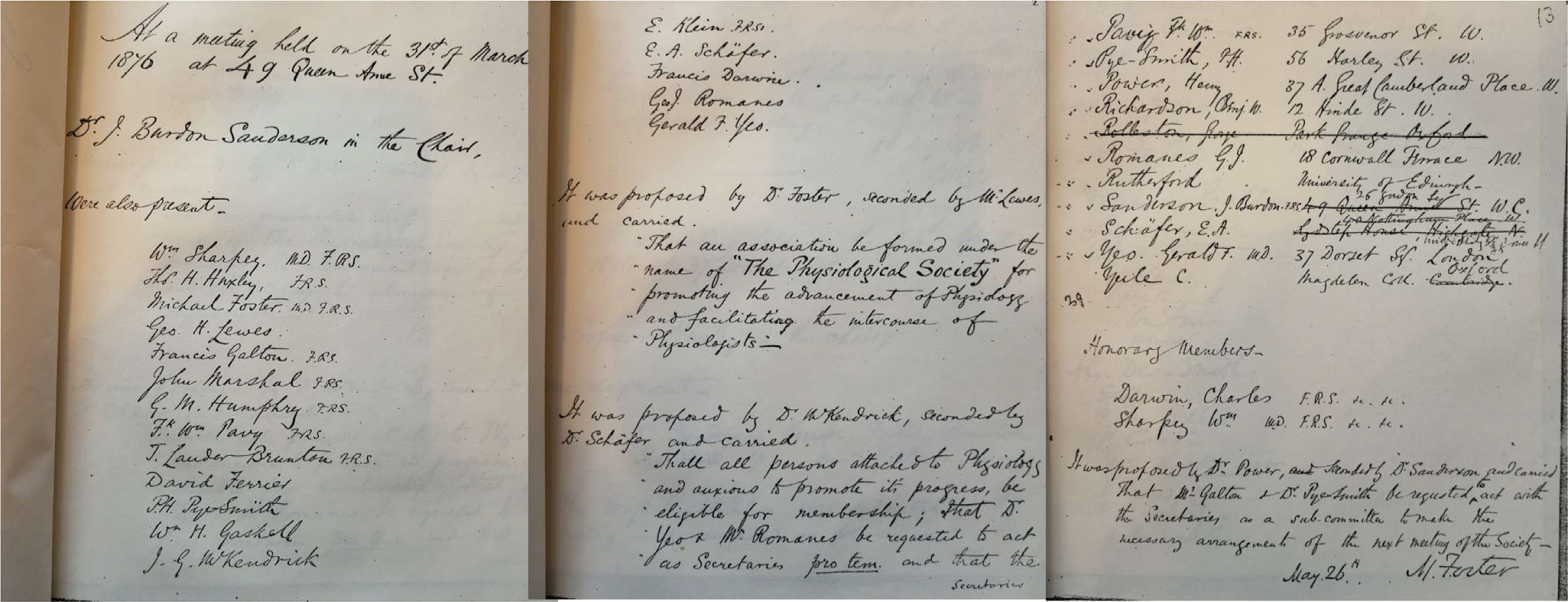
photographs of pages from the 1876 Minute Book showing that Burdon‐Sanderson chaired the inaugural meeting at which T. H. Huxley, Michael Foster and George Romanes were all present, and that Charles Darwin was elected one of the first Honorary Members

Further evidence for the close professional relations between Darwin and the early British physiologists comes from the minutes of the foundation meetings of The Physiological Society in 1876. The two titans of evolutionary biology, Charles Darwin and Thomas Henry Huxley, were foundation members. Figure 1 shows that the first meeting was chaired by Burdon‐Sanderson at his London home with Huxley, Foster and Romanes all present as founding members. The minutes also show Charles Darwin elected to Honorary Membership at the subsequent meeting in Romanes's home, when the minutes were signed by Michael Foster. When I first noticed these minutes during the Centenary celebrations of the Society in 1976 (Noble, 1976) I imagined that the founders simply wished to honour Charles Darwin as the greatest naturalist of the 19th century. I did not realise that the honour was also due to Darwin in his additional role in the science of physiology itself. Darwin clearly saw physiology as an essential cornerstone of the nuanced version of evolutionary theory that he was developing with Romanes in his last decade.

With this historical introduction, I will now turn to the role of physiology in evolutionary biology today and how it can vindicate Darwin's ‘beloved child’. I will show how we can echo Darwin's and Romanes's search for a physiological understanding of the evolutionary process and so complete Darwin's dream.

## 3 PHYSIOLOGY UNDERMINES THE FOUNDATIONS OF THE MODERN SYNTHESIS

### 3.1 Origin of the 2013 *Experimental Physiology* article

A decade ago, in 2012, I lectured to the Congress of the Chinese Association of Physiological Sciences in Suzhou (see video on https://www.youtube.com/watch?v=kOKOacjdi40), which was repeated as the President's Lecture at the 2013 International Congress of Physiological Sciences in Birmingham, UK, and subsequently published in *Experimental Physiology* (Noble, 2013). That article has been highly cited, but it, and particularly the videoed lectures on which it is based, were also the subject of a wave of abusive critical comments on social media and weblogs challenging all the evidence presented for physiology ‘rocking the foundations of evolutionary biology’ (see 2016 video on https://www.youtube.com/watch?v=KeVlBFX0qVc). Yet, over the intervening decade, there has been no response published in a peer‐reviewed journal by any of the vociferous critics. So, the article still stands and it is worth summarizing the central points. They were:

- Selection is at the level of organisms, not genes.
- Acquired characteristics can be inherited, as Darwin also assumed.
- There is no replicator separate from the vehicle.
- Genomes are not isolated from the organism and its environment.

**FIGURE 2:**
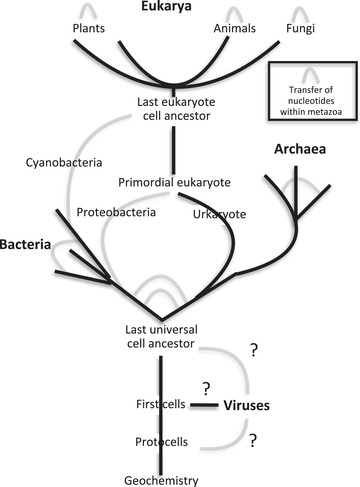
The tree of life becomes a network. Black lines represent the classical tree; grey lines represent the extent to which the tree has become a network There is promiscuous exchange and development of nucleotide sequences between unicellular life forms. Later symbiotic fusions of cyanobacteria and proteobacteria enabled the development of multicellular plants and animals. In multicellular organisms, plants, animals and fungi, the exchange of nucleotide sequences and proteins between somatic and germline cells can influence the development of future generations. (Based on the work of Carl Woese, who identified the Archaea, and a diagram from Franklin Harold, *In Search of Cell History*, University of Chicago Press, 2014)

There was sufficient evidence in 2013 to justify these points and that they require a fundamental revision of 20th century evolutionary theory which, incidentally, would bring it into line with Darwin's own later position. Selfish Gene theory (Noble, 2011) and the associated ideas of genetic causation (Noble, 2008a) need revising. One way to illustrate that need is to ask how the concept of the Tree of Life has developed. As illustrated in Figure 2, the tree idea as first sketched by Lamarck (1809) and Darwin (1837) has now become an extensive network as much as it is a tree.

### 3.2 Species have frequently exchanged their components during evolution

The reason the tree has become an extensive network is that organisms have frequently exchanged their components, including nucleotide sequences, during their evolution. Several papers and books published recently document the details (Noble, 2021a, 2021b, 2022; Shapiro, 2022a, 2022b; Shapiro & Noble 2021). Shapiro & Noble (2021) document in detail the many experimental discoveries made over a period of many years that fall outside the range of the M.odern Synthesis, but are neglected or downplayed in modern textbooks and popularisations. Out of 40 such discoveries identified and referenced, only three are given any attention at all, and usually not accurately. Many of these discoveries relate to the way in which species exchange, develop and fuse their nucleotide sequences and genomes (the grey lines in Figure 2).

Symbiogenesis (the process most relevant to Darwin's and Romanes's efforts), for example, is hardly referred to at all in Futuyma and Kirkpatrick's (2018) standard textbook, *Evolution*, and its main champion, Lynn Margulis, is not even openly acknowledged. Yet, as Darwin would surely have recognised given its proximity to the processes he was researching with Romanes, symbiogenesis was a crucial evolutionary transition, creating greatly improved production of ATP, leading to the possibility of multicellular organisms. Metazoan life as we now know it on our planet, including humans, simply would not have been possible without this transition. Plants developed from fusion of cyanobacteria with eukaryote ancestors to generate what became plastids. Alphaproteobacteria fused with urkaryotes to form mitochondria in the eukaryotic cell line.

Darwin and Romanes were therefore correct to look for physiological processes by which different species could fuse their constituent components and properties. Today, we know that this has happened time and again during the evolution of life on earth.

Had Romanes lived to witness the work of Mereschkowsky (1910) and Kozo‐Polyansky (1924) on the fusion processes that gave plants their energy‐producing plastids, he would have had the clue he needed: the first indications that fusion of different species could succeed in generating new species. The 20th century development of evolutionary biology could have been based on Romanes's idea of physiological selection, meaning selection of a fusion process that resulted in new physiological processes. Instead, we had to wait until 1971 for Lynn Margulis (1970, 1981) to show that a similar process had generated mitochondria in eukaryotes.

### 3.3 The hardening of the Modern Synthesis

Noble (2021a) complements the article with Shapiro (Shapiro & Noble, 2021) since it unravels the historical process by which orthodox evolutionary biology became trapped in a highly restricted version of the Modern Synthesis. The evolutionary biologist Steven J. Gould (2002) called this historical change the ‘hardening’ of the Modern Synthesis. That hardening has recently been analysed from a historical perspective in Noble and Noble (2022), showing that it can be dated to around 1963, when Julian Huxley wrote an Introduction to the second edition of his book *Evolution: The Modern Synthesis* (Huxley, 1942, 2010). Huxley's original book, the 1942 edition, was extraordinarily broad, with a substantial number of the discoveries identified by Shapiro and Noble (2021) acknowledged or foreseen. By contrast, the introduction to the 1963 edition is deeply influenced by the work of Watson and Crick on the double helix nature of DNA. Huxley writes:

I have left to the end the most important scientific event of our times – the discovery by Watson and Crick that the deoxyribonucleic acids – DNA for short – are the true physical basis for life, and provide the mechanism of heredity and evolution. Their chemical structure, combining two elongated linear sequences in a linked double spiral or bihelix, makes them self‐reproducing, and ensures that they can act as a code, providing an immense amount of genetical 'information,’ together with occasional variations of information (mutations) which also reproduce themselves. Linear constructions of DNA are, of course, the primary structures in the genetic organelles we call chromosomes. (Huxley, 1963, p 614 in the 2010 reprint)

This is the smoking gun in the story. In common with many other biologists at that time, Huxley was so impressed with the molecular biological discoveries of Watson and Crick and their interpretation as supporting the Central Dogma of molecular biology (Crick, 1958, 1970) that he did not stop to ask the question whether it really is true that the double helix ‘makes them self‐reproducing’, nor whether they really ‘act as a code’. Neither of Huxley's conclusions are correct. I am certainly not the first to point out the errors involved. Yet they are still not widely acknowledged.

### 3.4 Summary of why DNA does not self‐replicate

The essence of this argument can be summarized in five stages:

- DNA cannot replicate ‘like a crystal’ (Dawkins, 1976). It is a flexible thread wound around the chromatin proteins that can be partially unwound when it needs to be used as a template to make RNAs and proteins.
- The natural error‐rate of DNA replication is around 1 in 10^4^ which, in a genome of 3 billion base pairs, would generate as many as hundreds of thousands of errors.
- In normal cell division those errors are then corrected by the living cell which can reduce the error rate to just 1 in 10^10^.
- .Mismatches in the double helix, and other molecular clues, are used by the cell to enable the highly accurate error‐correction process.
- So far as we know, only the complex processes of a living cell make this possible.

Therefore, there is no replicator separate from its vehicle. DNA cannot replicate faithfully outside a living cell. This fact alone destroys Selfish Gene theory as a valid scientific hypothesis (Noble & Noble, 2022b).

### 3.5 ‘Selfishness’ in genes is not physiologically testable

Dawkins's justification for calling genes ‘selfish’ is that they increase their number in the gene pool and that this can be experimentally counted: ‘Genes can be counted and their frequency is the measure of their success’ (Dawkins, 2016, p 346). But this is vacuous since we cannot use the defining characteristic of a ‘selfish’ gene, that is, success in increasing its number in future generations, as the only experimental prediction the theory can make. The founding definitions of a valid theory cannot be used as experimental confirmation of the theory, since they are necessarily true. Nor can the problem be side‐stepped by defining all genes as selfish, as I earlier wrote:

What does ‘selfish’ mean in the selfish gene story? First we must decide whether ‘selfish’ defines a property that is universal to all genes (or even all DNA sequences) or whether it is a characteristic that distinguishes some DNA sequences from others. This is not as easy as it may seem. I suspect that the original intention was that all genes could be represented as ‘seeking’ their own success in the gene pool, regardless of how effective they might be in achieving this. One reason for thinking this is that so‐called junk DNA is represented in the selfish gene story as an arch‐example of selfishness: hitching a ride even with no function.

But on that interpretation, the demonstration that the concept is of no utility in physiological science is trivially easy. Interpreted in this way, a gene cannot ‘help’ being selfish. That is simply the nature of any replicator. But since ‘selfishness’ would not itself be a difference between successful and unsuccessful genes (success being defined here as increasing frequency in the gene pool), nor between functional and non‐functional genes, there would be no cashable value whatsoever for the idea in physiology. Physiologists study what makes systems work. It matters to us whether something is successful or not. Attributing selfishness to all genes therefore leaves us with nothing we could measure to determine whether ‘selfishness’ is a correct attribute. As metaphor, it may work. But as a scientific hypothesis it is empty. (Noble, 2011, p. 1010).

### 3.6 Physiological sensing and communication networks control the error‐correcting process

The fact that DNA is not a self‐replicator is what gives living organisms control over the error‐correcting process. The immune system uses this control to reduce error‐correction in the variable part of the DNA template for immunoglobulins and so generate millions of new DNA sequences from which the organism selects the very few that can work as the template for a successful antibody. The same process of hypermutation occurs in bacteria (e.g., in reaction to antibiotics) and in many other organisms when under stress. Organisms can therefore, at least partly, direct their own evolution. These are the reasons why evolution cannot be completely blind (Noble & Noble, 2017). Organisms have the ability to feel their way forwards in difficult times, which is when they employ hypermutation and other genetic processes to find a way through. The process is one in which disorder, such as random mutations, can be harnessed to serve the ordering regulatory processes in living systems (Noble, 2016; Noble & Noble, 2018).

Controlling the error‐correcting process is a well‐documented way for organisms to react functionally since, in the immune system, it is functionally directed for two reasons. First, the process is activated in response to environmental challenges, and is therefore targeted at meeting those challenges. Second, it can be targeted at specific sequence regions in the genome (Odegard & Schatz, 2006). Understanding the ability for organisms to achieve such targeting depends on unravelling the extraordinary processes by which events at the cell surface can trigger messages travelling via the microfilaments to specific regions of the nucleus (e.g., Ma et al., 2014; Kar et al., 2016). So much for the idea that the genes are ‘sealed off from the outside world’ (Dawkins, 1976). On the contrary, they are the most open to influences from the environment (Noble & Noble, 2021). For a valuable review of the physiological mechanisms of stress‐induced evolution see Mojica and Kueltz (2022), who list the five stress‐induced changes as: (1) mutation rates, (2) histone post‐translational modifications, (3) DNA methylation, (4) chromoanagenesis and (5) transposable element activity.

I will return to the role of signalling via microfilaments in a later section.

### 3.7 Are extracellular vesicles capable of functioning as Darwin's gemmules?

Extracellular vesicles (EVs) were first identified using electron microscopy. Cells were found to be surrounded by a variety of what appeared to be debris, ‘cellular dust’ (Corbel & Lorico, 2019). They are known to be formed by cells in a variety of ways. They are called exosomes when formed from multi‐vesicular bodies in cells, ectosomes or micro‐vesicles when formed from the cell membrane, and apoptotic bodies when released during cell death. Raposo et al. (1996) were the first to show that exosomes could contain components that induce T cell responses. Since then, functional properties have been found in a wide variety of clinical conditions, summarised in *Exosomes: A Clinical Compendium* (Edelstein et al., 2019). I was one of the editors of that volume and I was surprised by the wide variety of cell types and forms of communication that had been found in many different clinical conditions. It was impossible to avoid an obvious question. Darwin in 1868 had written ‘each cell casts off a free gemmule, which is capable of reproducing a similar cell’. His text only needs revising to read *capable of influencing other cells* (instead of ‘reproducing a similar cell’) for his gemmules to become the extracellular vesicles of today. After all, his idea did not need them to reproduce, only to influence characteristics. I therefore contributed an article myself to the book (Noble, 2019) drawing attention to the possibility that EVs and exosomes could function as Darwin's supposed gemmules.

### 3.8 Transmission of regulatory molecules and nucleotide sequences to the germline

Molecules capable of influencing gene regulation can be transmitted to the germ cells in a variety of circumstances, including in vitro transfers in which sperm cells act as vectors for introducing DNA into egg cells, transmission of regulatory small RNAs from the epididymus to epididymal spermatozoa, long distance transmission from the brain to the germline, and reverse transcription of nucleotide sequences into the genome (Cossetti et al., 2014; Chen et al., 2016; Chen, Yan & Duan, 2016; Lavitrano et al., 1989, 2006; Noble, 2019; Skvortsova et al., 2018; Spadafora, 2018; Zhang et al., 2018).

Good examples of functional transmission of soma characteristics include the work of Zhang et al. (2018) identifying the nucleotide sequences that transmit paternally acquired metabolic disorders, and Toker et al. (2022) showing the transgenerational inheritance of sexual attractiveness in *C. elegans* via small RNAs and HRDE‐1. The review by Skvortsova et al. (2018) is particularly valuable since it covers a very wide field of work on transgenerational inheritance and a wide variety of possible mechanisms.

The question now, therefore, is not whether Darwin's idea was correct in supposing that gemmules (aka EVs) exist, and that the soma can influence the germline, but rather what transgenerational forms of inheritance are actively promoted. This is a new field of research and it is full of opportunities for physiological approaches to clarify (see Allis et al., 2015). As physiologists we have no difficulty with accepting the influence of parental transmission on the health and disease of their children. Gluckman and Hanson's book, *The Fetal Matrix: Evolution, Development and Disease* (2005), showed even 17 years ago that we already know that Darwin was correct both in accepting the existence of parental influences in inheritance, but also in recognising the importance of physiology in understanding the processes by which evolution is achieved.

**FIGURE 3:**
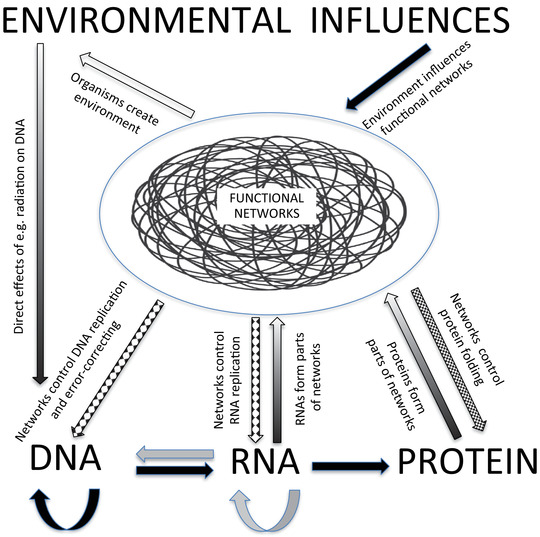
The Central Dogma of molecular biology (bottom row of the relations between DNA, RNA and proteins) placed in the context of physiological control by the functional physiological networks. Those networks are subject to environmental influences (black arrow) as well as contributing to the environment (white arrow). DNA expression and reorganisation is under control by the functional networks (hatched arrow). RNAs and proteins form important components of the functional networks (upward shaded arrows), while the functional networks determine how protein amino acid chains are folded (downward arrow from networks to proteins). The physical environment also has direct effects on DNA, for example, through radiation breakage. (Edited from Noble, 2021a; Figure 2)

In view of the immense impact that the Central Dogma had on Julian Huxley and the unnecessary hardening of the Modern Synthesis, it is time that the diagrams of the Central Dogma should be updated to include the physiological processes that control DNA replication, expression and reorganisation. Figure 3 does that by placing the functional physiological networks in a central place in the chains of causes and effects between the environment, the organism, its DNAs, RNAs and proteins.

Figure 3 also represents the extent to which feedback control is involved in organisms, all the way from the environment to the genome. We owe this understanding to the application of control theory in physiology, pioneered by Claude Bernard in the 19th century and Walter Cannon in the 20th. Bernard can therefore be regarded as the first systems biologist (Noble, 2008b). His work may well have been known to Darwin since the founders of The Physiological Society in 1876 much admired Bernard. He referred to the ‘constancy of the internal environment’, but today we know that none of the regulated variables are strictly constant. Organisms need to balance the regulation of one variable against another. Organisms are not simple thermostats. Bernard's ‘constancy of the internal environment’ has therefore been replaced by processes that require much more complex decisions in balancing the regulation of one controlled variable against that of many others.

## 4 SUMMARY OF EXPERIMENTAL FINDINGS CONTRARY TO THE MODERN SYNTHESIS

The unravelling of the fundamental bases of the Modern Synthesis depends on the accumulation of contrary experimental evidence by many scientists during the last 100 years. In this section I will briefly summarise those findings that are relevant to the diagrams in Figures 2 and 3, and indicate who was responsible for them. In my experience many physiologists are unaware of the major changes that are underway in evolutionary biology and why those changes are very important for the future contributions physiology could make to those developments. The aim of this section of my paper is to point the way for physiologists to understand and catch up on knowledge of these important evolutionary processes and to propose areas for future research.

### 4.1 Symbiogenesis

The process by which symbiogenesis became recognised as a major step in understanding evolutionary biology is the subject of a short review by Gray (2017). Lynn Margulis was the scientist responsible for resurrecting an idea first proposed by Mereschkowsky (1910) and Kozo‐Polyansky (1924) for the cyanobacterial origin of plastids (chloroplasts) in plants. Margulis (1970, 1981) identified alphaproteobacteria as the origin of mitochondria in eukaryotes. The evidence depends on:

a confluence of data — biochemical, molecular, and cell biological, coupled with the characterisation in a group of eukaryotic microbes (the jakobid flagellates) of a gene‐rich mitochondrial genome that strongly resembles a shrunken bacterial genome — now provides a compelling case for a single, endosymbiotic, alpha‐protobacterial origin of mitochondria. (Gray, 2017, p 1286)

In the case of plants ‘a compelling case for an endosymbiotic origin has always been easier to make for the plastid than for the mitochondrion.’ Gray also points out that ‘there is clearly much more to be discerned’ (p. 1287). This is an open invitation for physiology and genomics to investigate these issues further. There is also the open question: which other organelles might have originated through symbiogenesis? Recall that the lipid membranous structures do not depend on DNA templates. They must have had origins independent of DNA. Furthermore, the membranous structures of eukaryotic cells represent vast quantities of structural information which must be inherited in addition to DNA (Noble, 2017b). Lipid membranes are also the true ‘crystal‐like’ replicators. Lipid molecules automatically insert themselves into membranes, which is how membranes grow between cell replication cycles.

This is a suitable point at which to note that all attempts to draw tree–network diagrams, such as Figure 2, are compromises. Just like maps, they should not be confused with what they aim to represent. We should not take even Woese's revision as sacrosanct (Vane‐Wright, 2017).

### 4.2 Discovery of archaea

Until the work of Carl Woese (Woese, 1967; Woese & Fox, 1977) it was generally assumed that there was a linear progression of early life forms before the evolution of eukaryotes. Woese's great achievements were to identify a distinct group, the archaea, as phylogenetically distant from bacteria, and to show that eukaryotic forms have more biochemical properties in common with archaea than with bacteria. These discoveries (Woese, Kandler & Wheelis, 1990) led to the three‐part early Tree of Life forming the basis of Figure 2. Woese was trained and worked as a biophysical biochemist, the first sequence‐based phylogeneticist, but I also regard him as a brilliant physiologist. In 2005 he published an article in *Current Biology* in which he wrote:

I see the question of biological organization taking two prominent directions today. The first is the evolution of (proteinaceous) cellular organization, which includes sub‐questions such as the evolution of the translation apparatus and the genetic code, and *the origin and nature of the hierarchies of control that fine‐tune and precisely interrelate the panoply of cellular processes that constitute cells*. It also includes the question of the number of different basic cell types that exist on earth today: did all modern cells come from a single ancestral cellular organization? (Woese, 2005, my emphasis)

He correctly saw the significance of ‘hierarchies of control that fine‐tune cellular processes’ (represented here in Figure 3), which can be viewed as a perspective very similar to the principle of biological relativity, that is, causation from and to all levels of organisation (Noble, 2016). His work was strongly resisted by evolutionary biologists adhering to the Modern Synthesis (Mayr, 1998).

### 4.3 Discovery of natural genetic engineering

The idea of ‘natural’ genetic engineering should be uncontroversial, yet it also has been strongly resisted. After all, what scientists now achieve in genetically engineering organisms is frequently based on the CRISPR techniques first discovered in prokaryotes, endowing them with the natural biochemical processes that form their equivalent of the immune system by generating acquired resistance to viruses (Barrangou et al., 2007). This work led to the award of the Nobel Prize in Chemistry to Charpentier and Doudna in 2020.

But the idea that organisms can themselves engineer changes in their nucleotide sequences and change the organisation of their genomes originates much earlier with the work of Barbara McClintock who, in the 1940s and 1950s, showed that maize plants reorganise their chromosomes when under stress. As early as the 1930s she showed the link between chromosomal rearrangement and the recombination of genetic traits. Julian Huxley knew about similar work in his book *Evolution: The Modern Synthesis* (Huxley, 1942; see Huxley, 2010, p. 137). Yet, when McClintock (1953) published in the journal *Genetics* she was completely ignored. Three decades later (1983) she was awarded the Nobel Prize in Physiology and Medicine. In her Nobel lecture (McClintock, 1984) she clearly enunciated the principle that the physiology of cellular control is the key in understanding these phenomena.

McClintock's mantle was then inherited by James Shapiro, a bacterial geneticist at the University of Chicago, who demonstrated the process of genetic engineering and reorganisation of genomes in bacteria (Shapiro, 1992, 2011, 2022a, 2022b). This major transformation of the molecular biology of evolutionary processes has also been strongly resisted by supporters of the Modern Synthesis, since his work involves non‐random and saltatory mutations as well as the violation of the Central Dogma that protein action cannot change the genome (and possibly because Shapiro has repeatedly described these capacities of organisms as a form of intelligence). He is in good company since Darwin also used ‘intelligence’ to characterise the capacities of worms and plants (Bradley, 2020, pp. 63–67). The refusal by many evolutionary biologists to recognise how control processes in living systems form the basis of intelligence is a deep misunderstanding of evolution. No‐one doubts that humans and other primates show what we naturally call intelligence. Yet their, and our, intelligent abilities must themselves have evolved from other organisms, including single cell organisms. Evolution has generated those processes naturally through successive transitions, each of which enables further transitions with new characteristics. Those processes are properties of living organisms and are proper subjects for physiological research since stochasticity in living organisms is harnessed (used) by physiological control processes (Noble & Noble, 2018, 2022a). Shapiro's work is now beautifully collated in the latest edition (Shapiro, 2022a) of his book *Evolution: A View from the 21st Century*.

The use of the word ‘natural’ here is comparable to the distinction Darwin made between natural and artificial selection. In his 1859 book, Darwin invented the idea of natural selection by comparison with deliberate (artificial) selection by humans breeding animals and plants for desirable characteristics. But he also realised that the same deliberation is manifest in the choices (sexual selection) made by many organisms, including birds (Darwin, 1859; 1868, vol. 2, pp. 75; 1871, chap. 8).

### 4.4 The tree becomes a network

In addition to the processes of natural genetic engineering, living organisms have been promiscuous in the exchange and reorganisation of nucleotide sequences. It was formerly thought that such exchange is limited to single‐cell organisms but, as discussed earlier, cells in multicellular organisms also convey nucleotide sequences to each other via extracellular vesicles.

Darwin is justly acknowledged for his famous ‘I Think’ tree sketch in one of his experimental notebooks (Darwin, 1837), though it should be more widely known that Lamarck first drew a tree of life nearly three decades earlier in 1809 (see Gould, 2000). I doubt whether either Darwin or Lamarck would be surprised that their 19th century attempts to capture the evolutionary connectedness of all species should now be supplanted by a tree–network, as in Figure 2. Both were flexible in the light of evidence, Darwin through his gemmules idea, leading to acknowledgement that natural selection is not the only process in evolution, and Lamarck through abandoning his original idea of a single ladder of life.

Yet, when the British Magazine *The New Scientist* published an editorial (Anon, 2009) on this seemingly obvious and important development, it was immediately greeted with derision (Dennett et al., 2009): ‘First it's false, and second, it's inflammatory.’ Why? Because ‘Your cover was handing the creationists a golden opportunity.’ I have some sympathy for this problem since I have myself been misrepresented by creationists. But we should be answering misrepresentation by patiently explaining the correct interpretation. Scientists should not be seeking to close down debate and discussion. Incidentally, Dennett et al. accepted that the tree has now become a network, but then downplayed the fundamental significance of interspecies and transgenerational transmission of nucleotide sequences and characteristics:

Of course there's a tree; it's just more of a banyan than an oak at its single‐celled‐organism base. The problem of horizontal gene‐transfer in most non‐bacterial species is not serious enough to obscure the branches we find by sequencing their DNA.

This is the kind of reasoning that led supporters of the hardened version of the Modern Synthesis to strongly oppose Carl Woese's use of nucleotide sequencing of bacterial and other species to discover the archaea, leading to the significance of the processes of symbiogenesis. Playing down the significance of important discoveries hinders adventurous research by pretending that ‘nothing much/fundamental has changed’. Not for nothing was Carl Woese described in *Science* as ‘microbiology's scarred revolutionary’ (Morell, 1997). Furthermore, unicellular life forms are by far the most numerous and probably responsible alone for 1–2 billion years of evolutionary history, while lateral transfer between cells in metazoa and plants is precisely what enables the inheritance of Lamarckian‐style use‐and‐disuse characteristics in species with specialised germlines.

### 4.5 Communication between membrane receptors and nuclear DNA

The discovery of the functional significance of extracellular vesicles is not the only example in modern physiological research where the revolution in resolution in microscopy matters. The ability to visualise the extensive networks of fine filaments in living cells using fluorescent marking has also provided a solution to another problem in evolutionary biology: if organisms can manipulate their nucleotide sequences in ways that react functionally to environmental stress, how do nuclear components react to external influences sensed by the cell membrane receptors? The answer is that sub‐membranous changes, for example, in ion concentrations due to the opening of ion channels, trigger molecular messages that can travel on the molecular motors moving along the microfilaments and so travel to specific locations in the nucleus.

To visualise this, imagine a small protein around 1 nm in radius located near the cell membrane. The nucleus of a small cell around 20 μm in size would therefore be around 10 μm from the surface membrane. If we magnified the small protein to be around 1 cm (as it might be sketched in a diagram), a magnification of 10 million times, the nucleus would appear to be 100 km away, roughly the distance from Oxford to London. For a large cell around 100 μm, such as a human oocyte, the nucleus would appear to be 1000 km away, roughly the distance to the far north of Scotland. The microfilaments that transport the motors and their cargo are about 25 nm in diameter and, on the same magnification would be the size of a small footpath running the whole length of the country.

Yet accurate and targeted transport of messenger molecules over these tiny cell ‘roadways’ has been discovered in living cells. Examples of recent physiological studies that demonstrate this process can be found in the papers of Ma et al. (2014) and Kar et al. (2016), working on the transmission of signals from calcium concentration changes that control the relevant gene activity in the nucleus. The molecular motors can achieve this transport at a speed of up to 2 μm/s. The nucleus can therefore be reached within just a few seconds. Visualising these processes using fluorescent markers reveals a vast trafficking system with messenger molecules moving rapidly in all directions between the cell and its nucleus. The work of Kar et al. (2016) is ground‐breaking in showing the dependence on *two* calcium compartments. Multiple causation must surely be the norm in physiological control systems.

These studies open the way for many further physiological investigations on how cells control their genomes, and so may make major contributions to evolutionary biology. Barbara McClintock predicted in 1984 that the genome would be found to be: ‘an organ of the cell, monitoring genomic activities and correcting common errors, sensing the unusual and unexpected events, and responding to them by restructuring the genome’ (McClintock, 1984). Physiology is now in a position to fulfil her dream too. Discovering the cellular signalling pathways that can regulate gene expression and proof‐correcting of DNA replication would be crucial to fulfilling that dream.

### 4.6 Lamarckian forms of inheritance

The French biologist Jean‐Baptiste Lamarck was professor of natural history of insects and worms at the Botanical Garden in Paris when he published his great work on evolution, *Philosophie Zoologique*, in 1809. He investigated natural processes by which evolution could have occurred. One of these was the physiological process of use and disuse. In modern physiology, that process is evident everywhere in the body. Identical twins who choose different lifestyles naturally develop different muscular structure, and physiologists have now identified the RNAs that mediate differential expression of muscle proteins (Bathgate et al., 2018). A crucial evolutionary question now is whether and how those control characteristics can be transmitted across generations. This work provides a specific goal for research on physiological signalling, particularly because it would ideally require identification of multiple causation pathways, since many genes are involved in the use–disuse regulation of muscle proteins (Ahmetov and Fedotovskaya, 2015). The association levels with individual genes are very low.

When Lamarck wrote his book he also thought, initially, that the process of increasing complexity of life could be represented as a ladder of life, continuous with no branching. But, as I have already noted, he replaced this concept with his drawing of the first Tree of Life (Lamarck, 1809, 1994, p. 649 in 1994 reprint). Lamarck's tree of life is much more detailed than Darwin's sketch.

For championing evolution by natural processes he was praised by Darwin as ‘this justly celebrated naturalist … who upholds the doctrine that all species, including man, are descended from other species’ (Darwin, 1869). But in his own time in Paris he was completely trashed by his arch rival at the natural history museum, Georges Cuvier, who was a serial creationist. When Neo‐Darwinism grew in ascendance in the early 20th century, based on eliminating the inheritance of acquired characteristics from evolutionary biology, Cuvier's ridicule was echoed by those who developed the Modern Synthesis. Lamarck's reputation as ‘this justly celebrated naturalist’ has never recovered.

Yet, there is ample evidence that Lamarck was essentially right (Allis et al., 2015; Bateson & Gluckman, 2011; Escobar et al., 2021; Gissis & Jablonka, 2011; Gluckman & Hanson, 2005; Gluckman et al., 2016; Jablonka, 2016; Jablonka & Lamb, 2005, 2014; Noble, 2021b; Skvortsova et al., 2018). The demise of the Weismann Barrier, following the discovery that regulatory nucleotide sequences developed by soma cells can be transmitted to the germline, resurrects the valid question: how many such characteristics are transmitted in this way?

There are two factors standing in the way of research on this question. The first is that few funding agencies are currently likely to accept proposals. We must hope that will change with time as people become more aware of the changes that are rapidly developing in the field of evolutionary biology. The second is the multi‐genic nature of physiological control. As I have already noted in the work of Kar et al. (2016), identifying *multiple* pathways of gene regulation is challenging, but forms an essential part in unravelling the physiological control processes involved.

### 4.7 Demise of gene‐centrism

Gene‐centric interpretations of physiology and evolution are far from achieving their goals. One reason for this impasse is that association studies do not reveal physiological causation (Felin et al., 2021a, 2021b). With Peter Hunter I have recently outlined how this impasse might be negotiated (Noble & Hunter, 2020). Modelling physiological regulatory networks could help to explain the low association scores and identify where causation exists even when the association score is very low. It all depends on how robust the networks are and how easily they can switch from one pathway to another.

The details on why we need to move on from Selfish Gene theory, as popularised by Dawkins (2016, 1976), have been published in Noble and Noble (2022b). *The Selfish Gene* was a brilliant popular exposition of Neo‐Darwinism, but moving away from its simplicity is essential for the future of physiology and evolutionary biology. Dawkins himself has stated that ‘in some ways I would quite like to find ways to recant the central message of *The Selfish Gene*. So many things are fast happening in the world of genomics….’ (Dawkins, 2016, p 345). Indeed they are, and I believe he can.

### 4.8 Function, purpose and teleology

The purposive teleological language used in some parts of this article is deliberate. But I recognise that most scientists, including many physiologists, have been trained, as I was, to avoid such language in favour of a passive descriptive form. I now use purposive language because I think that the existence of purpose in organisms is a proper object of physiological study, as argued in a recent article with my brother, Raymond (Noble & Noble, 2022a). Living organisms are naturally purposive. They must use anticipation and creativity in behaviour to survive. The physiological processes involved must therefore have evolved. How purposive anticipatory behaviour can be explained physiologically and how explanations based on it can be tested empirically are the main foci of some of our recent articles. Here I briefly summarise the main conclusions.

1. The harnessing of stochasticity (first referred to in Noble, 2017a and extensively developed in Noble et al., 2019; Noble & Noble, 2017, 2018, 2021, 2022a, 2022b) is a necessary process since, if chance is merely experienced (the Neo‐Darwinist view) rather than used functionally, the faculty of choice is not possible. Purposive behaviour depends on that faculty. Without it, organisms would be automata. Purely passive descriptions of their behaviour would then suffice.
2. Organisms capable of choice exhibit unlimited associative learning, which is one of the empirical criteria for being able to attribute consciousness and deliberative anticipatory action (Ginsburg & Jablonka, 2019). Using that criterion those authors date the evolution of this faculty as around the time of the Cambrian Explosion, c. 500 million years ago, in which case it vastly predates the evolution of the human species, and must be more widespread than commonly assumed.
3. The unlimited nature of such learning also precludes representation of organisms with agency as following specific fixed algorithms. Fixed algorithms cannot generate behaviour dependent on the harnessing of stochasticity, since specific outcomes are then necessarily unpredictable, although they may be explicable in retrospect. The behaviour is more comparable to a game in which the participants alter the rules as the game progresses (see also item 6). Yet those flexible rules govern what happens.
4. The processes of choice in organisms with nervous systems may include neuronal circuits that are subject to neural selection, as first proposed by Gerald Edelman (1978) (and see Noble & Noble, 2021 for explanation). Edelman's idea was summarily dismissed by Crick (1989) as incompatible with Neo‐Darwinist interpretations of evolution, which led to its neglect. This is yet another opportunity for physiological research, specifically neuroscience, to contribute to evolutionary biology. It is also an example of how the Neo‐Darwinist mind‐set restricts the questions that are regarded as valid. Crick's dismissal of Edelman's Neuronal Selection theory was based on the requirement of a strict separation between replicator and vehicle. Edelman's idea did not require that. Nor does such separation exist, even for the genome.
5. The forms of causation differ in important ways between the various levels of organisation in living organisms (Noble et al., 2019). Most relevant to the question of agency and purpose, social factors have a primary role, as explained in Noble and Noble (2021) and in Noble and Ellis (2022). In principle, it is now possible to understand how immaterial social factors can play the role they must if agency is to be possible. Most importantly, it is not necessary to resort either to Cartesian dualism or to supernatural events to provide an explanation.
6. There is current interest in whether the development of artificial intelligence (AI) could achieve the criteria for the equivalent of agency in living organisms (Noble & Noble, 2019). In those discussions Raymond and I have suggested that this may be difficult or even impossible with silicon‐based materials. To the extent that a living organism can be compared to a computer (Bray, 2011), organisms are aqueous ‘computers’, with access to a vastly greater degree of stochasticity at the molecular level. A significant challenge for AI research is whether it would be necessary to develop water‐based computational systems. It took evolution billions of years to do that. I doubt whether the achievement of agency in AI systems is just around the corner.

The issue of agency and purpose in organisms is still strongly disputed in evolutionary biology. However, with the exception of agency itself, the majority of the possible research opportunities for physiology's future contributions to evolutionary biology outlined in this article do not depend directly on this issue. Readers who prefer to reject the idea of agency may still find valuable ideas for research in what I have outlined.

## 5 CONCLUSION

I would like to think that Charles Darwin would be delighted that, over a century later, his links with physiology through his work with Burdon‐Sanderson and with Romanes have been spectacularly reborn. His dream is now very much alive. It is time for physiology to come to the rescue of evolutionary biology by providing the evidence for the causal mechanisms of evolutionary change, which Darwin himself believed was lacking from his theories (Bradley, 2022; see also West‐Eberhard, 2008), and which are still lacking from the standard theories today.

During the writing of this article, Denis Noble became a Fellow of the Linnean Society.

## COMPETING INTERESTS

None.

## AUTHOR CONTRIBUTIONS

Sole author.

## FUNDING INFORMATION

None.
